# Conversion of t11t13 CLA into c9t11 CLA in Caco-2 Cells and Inhibition by Sterculic Oil

**DOI:** 10.1371/journal.pone.0032824

**Published:** 2012-03-12

**Authors:** Anne-Catherine Schneider, Pauline Beguin, Sophie Bourez, James W. Perfield, Eric Mignolet, Cathy Debier, Yves-Jacques Schneider, Yvan Larondelle

**Affiliations:** 1 Institut des Sciences de la Vie, Université Catholique de Louvain, Louvain-la-Neuve, Belgium; 2 University of Missouri, Columbia, Missouri, United States of America; Max Planck Institute for Chemical Ecology, Germany

## Abstract

**Background:**

Conjugated linoleic acids (CLA), and principally c9t11 CLA, are suspected to have numerous preventive properties regarding non-infectious pathologies such as inflammatory diseases, atherosclerosis and several types of cancer. C9t11 CLA is produced in the rumen during biohydrogenation of linoleic acid, but can also be synthesized in mammalian tissues from *trans*-vaccenic acid (C18:1 t11) through the action of delta-9 desaturase (D9D). For several years, it is also known that c9t11 CLA can be synthesized from conjugated linolenic acids (CLnA), *i.e.* c9t11c13 CLnA and c9t11t13 CLnA. This study aimed at investigating to which extent and by which route c9t11 CLA can be produced from another isomer of CLA, the t11t13 CLA that is structurally very similar to c9t11t13 CLnA, in Caco-2 cells.

**Methodology/Principal Findings:**

Caco-2 cells were incubated for 24 h with 20 µmol/l of t11t13 CLA in the absence or presence of sterculic oil used as an inhibitor of D9D. Caco-2 cells were able to convert t11t13 CLA into c9t11 CLA, and c9t11t13 CLnA was formed as an intermediate compound. In the presence of sterculic oil, the production of this intermediate was decreased by 46% and the formation of c9t11 CLA was decreased by 26%. No other metabolite was detected.

**Conclusions/Significance:**

These results not only highlight the conversion of t11t13 CLA into c9t11 CLA but demonstrate also that this conversion involves first a desaturation step catalysed by D9D to produce c9t11t13 CLnA and then the action of another enzyme reducing the double bond on the Δ13 position.

## Introduction

For about thirty years, numerous studies have focused on conjugated linoleic acids (CLA) and their beneficial effects on animal and human health. CLA is a collective term to describe a class of geometric and positional isomers of linoleic acid, essentially present in products derived from ruminants [1], [2]. The most abundant isomer of CLA is the *cis*-9, *trans*-11 CLA (c9t11 CLA), also named rumenic acid, which represents about 80–90% of the total CLA of dairy products [1]–[4]. Several studies have highlighted that c9t11 CLA has interesting properties that may improve human health. Pariza & Hargraves [5] first reported the anticarcinogenic properties of partially-purified extracts from grilled ground beef. Later, many other authors demonstrated that CLA has beneficial effects regarding the prevention against some cancers *in vivo* [6]–[8] and inhibits cell proliferation of cancer cells *in vitro* [9]–[11]. In addition, CLA has also shown anti-atherosclerotic [12], [13] and anti-inflammatory [3], [14], [15] properties.

The c9t11 CLA found in the fat of dairy products originates from two distinct sources. The first origin of c9t11 CLA is its formation during the ruminal biohydrogenation of linoleic acid [2]–[4]. C9t11 CLA can thus be viewed as an intermediate compound produced during the microbial conversion of linoleic acid to stearic acid. Some c9t11 CLA escape from ruminal biohydrogenation, are absorbed at the intestinal level and can be transferred to the animal tissues and the milk produced. The second origin of c9t11 CLA is synthesis within the tissue from *trans*-vaccenic acid (C18:1 t11, TVA), another important intermediate compound produced during the biohydrogenation process, which also partly escapes from the rumen before being absorbed by the intestine. This endogenous synthesis of c9t11 CLA from TVA is due to the action of delta-9 desaturase (D9D). It occurs in lactating cows [16]–[18] as well as in humans [19], mice [20], cultured MCF-7 and SW480 cells [21], and represents the primary source of tissular c9t11 CLA.

Alternative biohydrogenation pathways lead to the production of a whole range of fatty acid metabolites including additional CLA isomers. T11t13 CLA is one of the minor isomers found in dairy fat [22]. Its level can however be increased by a supply of high linolenic acid oil to dairy cows [23], [24]. A recent study showed that the t11t13 CLA isomer given to human intestinal HT-29 cells was partially converted to c9t11 CLA [25]. This very first study reporting the conversion of one isomer of CLA into another one did unfortunately not elucidate the biochemical pathway involved.

Conjugated fatty acids other than CLA are found in some seed oils. These are isomers of α–linolenic acid containing three conjugated double bonds and are named conjugated linolenic acids (CLnA) [26]. As an example, punicic acid (c9t11c13 CLnA) is found at up to 83% of total fatty acids in pomegranate (*Punica granatum*) seed oil and α–eleostearic acid (c9t11t13 CLnA) at up to 67.7% and 56.2% in tung and bitter gourd (*Momordica charantia*) seed oils, respectively [27]. It has been reported in the literature that these two CLnA, closely similar to the c9t11 CLA in terms of structure, can be converted into c9t11 CLA in rats, mice and humans when the corresponding oil was provided to the subjects [28]–[32]. In accordance with these *in vivo* data, we very recently observed the same conversions in Caco-2 cells used as an *in vitro* model of the human intestinal epithelium [33]. The consumption of CLnA gives thus another way than the dietary intake of dairy products to produce c9t11 CLA in mammals. It is therefore a third source of supply of this fatty acid.

**Figure 1:**
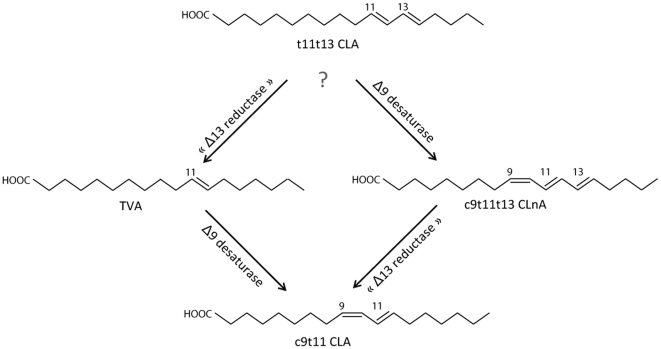
Hypothetical metabolic pathways of conversion of t11t13 CLA into c9t11 CLA in human intestinal Caco-2 cells.

In the present study, we aimed to determine the extent and through which biochemical pathway the isomer t11t13 CLA can be converted into c9t11 CLA in Caco-2 cells. With the exception of the *cis* double bond in Δ9 position, t11t13 CLA is structurally similar to c9t11t13 CLnA that can be converted into c9t11 CLA in Caco-2 cells by the reduction of the double bond in the Δ13 position [33]. Two alternative pathways can be proposed for the formation of c9t11 CLA from t11t13 CLA (Figure 1). Either, t11t13 CLA first undergoes a desaturation step catalysed by the D9D and is then reduced in its Δ13 position, or t11t13 CLA is first reduced in its Δ13 position and then becomes the substrate of the D9D. The intermediate compound formed would thus be c9t11t13 CLnA in the first case and TVA in the second case. To elucidate if one of these two proposed pathways takes place in Caco-2 cells, intermediates were measured in cells incubated with t11t13 CLA for 24 h. In addition, sterculic oil (SO) was used as an inhibitor of the D9D activity in order to modulate the accumulation of the intermediate compounds. Indeed, SO contains about 60% of sterculic acid [8-(2-octyl-1-cyclopropenyl) octanoic acid] and about 10% of malvalic acid [7-(2-octyl-1-cyclopropenyl) heptanoic acid], which are known to inhibit the D9D activity [34], [35].

## Materials and Methods

### Cell culture

Caco-2 cells were purchased from the American Type Culture Collection (ATCC; Rockville, MD) and were used between passages 43 to 51. Cells were cultured in a serum-free hormono-defined synthetic medium (Basal Defined Medium or BDM [36], [37]). This medium was composed of a 5∶5∶1 (v∶v∶v) mixture of Iscove's Modified Dubelcco's Medium (IMDM; Lonza, Verviers, BE), Ham's F12 medium (Lonza) and NCTC-135 (Invitrogen, Carlsbad, CA) supplemented with 1% (v∶v) non-essential amino acids (Lonza), 2 mmol/l L-glutamine (Lonza), 0.06 mmol/l ethanolamine (Sigma-Aldrich, St Louis, MO), 1 ng/ml epidermal growth factor (Sigma-Aldrich), 100 nmol/l hydrocortisone (Sigma-Aldrich), 2 nmol/l triiodothyrosin (Sigma-Aldrich), 1 µg/ml insulin (Sigma-Aldrich), 10 µg/ml albumin complexed to linoleic acid (Sigma-Aldrich) and 3 g/l NaHCO_3_ (Sigma-Aldrich). Caco-2 cells were maintained at 37°C in a humidified atmosphere made of 5% CO_2_ (v∶v) in air. Cells were sub-cultured after reaching 90% confluence by an HyQ-Tase solution (Perbio-Sciences, HyClone, Erembodegem, BE) and were seeded on type I collagen (1% in PBS, v∶v) (Sigma-Aldrich) precoated supports. After sub-cultivation, cells were seeded at a density of 3×10^4^ cells per cm^2^ in either 6-well plates or in 25 cm^2^ flasks and grown for 9 days with medium changes every 2 days.

### Lipid treatment

At 7 days post-confluence (9 days of culture in total), Caco-2 cells were treated with t11t13 CLA or TVA (Larodan, Malmö, SE) or with the ethanol vehicle (1%, v∶v; Sigma-Aldrich) in the presence of absence of SO (see section below) for 24 h. The fatty acids were first dissolved in ethanol and then added to the culture medium at a concentration of 20 µmol/l, in the presence of 1 mmol/l taurocholic acid (Sigma-Aldrich).

### Preparation of sterculic oil

SO was extracted from the seeds of the *Sterculia foetida* tree. Seeds were dehulled, finely ground, loosely packed into an extraction thimble, and then placed in a Soxhlet apparatus for extraction. Petroleum ether was refluxed through the apparatus for 8 h, and then collected and dried using a rotary evaporator to obtain the extracted oil. Extracted SO was then saponified basing on the saponification protocol of Guil-Guerrero & Belarbi [38] with adaptations in the amounts and volumes used. Briefly, about 120 mg of oil were mixed with 400 µl of a saponifying solution made of KOH (60 mg), water (200 µl) and ethanol (200 µl; 96%, v∶v). After 1 h incubation at 70°C, 100 µl water was added and unsaponifiables were separated by the addition of 1 ml of hexane (Biosolve, Valkenswaard, NL). After elimination of the upper phase (containing unsaponifiables), 240 µl of 1.2 mol/l HCL in water were added to acidify and 1 ml of hexane was then added to extract free fatty acids (FFA). After centrifugation, the hexane phase containing FFA was dried under nitrogen and reconstituted in 10 ml of ethanol. This solution of saponified oil in ethanol was then added to the culture medium in order to reach a final concentration of 300 µmol/l in sterculic acid, which is the most abundant fatty acid present in SO and has been shown, with others, to efficiently inhibit the D9D activity.

### Sample preparation and separation of lipid classes by Solid Phase Extraction

After 24 h of treatment, cells were washed, scrapped and collected in 1 ml of PBS. The cell suspension was then divided into two parts, one for lipid extraction and the other for protein content determination. Both parts were pelleted by centrifugation for 10 min at 500 g.

With the first part of the sample, total cell lipids were extracted with chloroform/methanol/water (2∶2∶1.8; v∶v∶v) (Biosolve) according to the Bligh & Dyer method [39]. Tridecanoic acid (C13:0; Sigma-Aldrich) was used as internal standard. Each sample was then dried under nitrogen and methylated at 70°C through the addition of 1 ml of 0.1 mol/l KOH in methanol and a 1 h incubation followed by the addition of 0.4 ml of 1.2 mol/l HCL in methanol and incubation during 15 min. The fatty acid methyl esters (FAME) were then extracted by 2 ml of hexane and separated by GC.

Between the extraction and methylation steps, the total lipid extracts of certain samples were separated by solid phase extraction (Bond Elut–NH_2_, 200 mg, 3 ml; Varian, Middelburg, NL) into 3 lipid fractions, *i.e*. neutral lipids (NL), FFA and phospholipids (PL). A mixture of three internal standards made of triheptadecanoin (Larodan), tridecanoic acid and 1,2-dipentadecanoyl-sn-glycero-3-phosphatidylcholine (Larodan) was added to each sample before lipid extraction in order to allow the quantification of the fatty acids present in the NL, FFA and PL fractions, respectively. After conditioning the columns with 3 ml of hexane, the total lipid extract dissolved in 200 µl of chloroform was loaded. After the chloroform was pulled through, the NL fraction was eluted with 1.8 ml of chloroform/2-propanol (2∶1, v∶v) (Biosolve). The column was then loaded with 2.4 ml of diethyl ether/acetic acid (98∶2, v∶v) (Bioslove) followed by 1.8 ml of methanol (Biosolve) to elute the FFA and PL fractions, sequentially. Each fraction was then dried under nitrogen and methylated as described above. The FAME were then extracted by 2 ml of hexane and separated by GC.

### Fatty acid analysis

The FAME obtained after extraction of cellular samples were separated by gas chromatography as described in Dang Van *et al.* [22]. Fatty acid peaks were identified using pure methyl ester standards purchased from Larodan and from Nu-Chek Prep, Inc (Elysian, MN).

**Figure 2:**
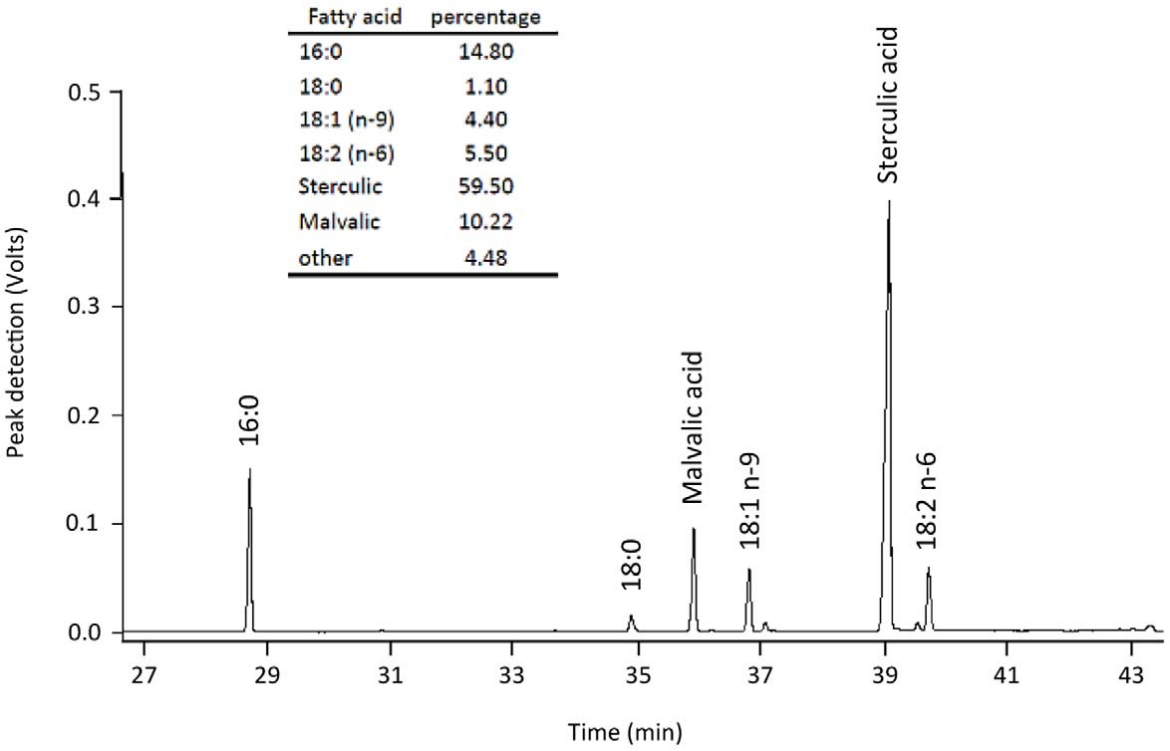
Representative gas chromatogram of sterculic oil extracted from the seeds of the Sterculia Foetidia tree. FAME were analysed using a gas chromatograph equipped with a SP2560 column (100 m×0.25 mm internal diameter, 0.2 µm film thickness). Gas chromatograph conditions were a flow rate of He of 1 ml/min with an initial temperature of 140°C held for 5 min. The column temperature was then increased to 250°C at a rate of 2°C.min-1, and then held at 250°C for 15 min.

The fatty acid composition of the oil extract (Figure 2) was also determined by gas chromatography. Briefly, the FAME were prepared by transesterification with sodium methoxide according to the method of Christie [40]. They were then analysed using a gas chromatograph (Varian Star 3400, Santa Clara, CA) equipped with a SP2560 column (100 m×0.25 mm internal diameter, 0.2 µm film thickness; Supelco, Bellefonte, PA). Gas chromatograph conditions were a flow rate of He of 1 ml/min with an initial temperature of 140°C held for 5 min. The column temperature was then increased to 250°C at a rate of 2°C.min^−1^, and then held at 250°C for 15 min. Fatty acid peaks were identified using pure methyl ester standards (Supelco).

### Protein determination

The samples prepared for the determination of cell protein contents (see above) were lysed in Reporter Lysis Buffer (RLB; Promega, Madison, WI). Protein concentration was then determined using the Bicinchoninic Acid Protein Assay kit (Sigma-Aldrich), which is based on the alkaline reduction of Cu^2+^ to Cu^1+^ by proteins followed by the formation of a purple complex of Cu^1+^ with bicinchoninic acid (BCA). 25 µl of cell lysate were mixed with 200 µl BCA working reagent. After incubation at 37°C for 30 min, the Cu^1+^-BCA complex formed was quantified at 600 nm with a spectrophotometer (SpectraCount™, Packard, Warrenville, IL). Protein concentrations were calculated using a bovine serum albumin (BSA; Sigma-Aldrich) calibration curve obtained for each assay with a known concentration range of BSA (0.2–2 mg/ml).

### Statistics

Data for intracellular fatty acid composition are reported as means from 3 independent experiments, each performed in triplicates. Data were subjected to an analysis of variance (ANOVA 1) using SAS (version 9.2; SAS Institute Inc., Cary, NC) coupled with the Tukey test in order to identify means with significant differences (*P* value <0.05).

## Results

### Accumulation and conversion of t11t13 CLA

We first observed that, when 7-days post confluent Caco-2 cells cultivated in serum-free medium were incubated during 24 h in the presence of 20 µmol/l of t11t13 CLA, they were able to absorb and accumulate this isomer of CLA (Table 1). Furthermore, these cells also converted t11t13 CLA into c9t11t13 CLnA and c9t11 CLA, these fatty acids being absent from control cells.

**Table 1:** Total fatty acid composition of Caco-2 cells.

	Control		Control+SO		t11t13 CLA^2^		t11t13 CLA+SO		TVA^3^		TVA+SO	
Fatty acids												
	Mean (ng/µg)	SD^4^	Mean (ng/µg)	SD	Mean (ng/µg)	SD	Mean (ng/µg)	SD	Mean (ng/µg)	SD	Mean (ng/µg)	SD
**C14:0**	12.8^d^	1.2	17.1^a^	1.1	14.2^c,d^	1.6	16.7^a,b^	1.5	14.9^b,c^	1.1	17.1^a^	1.6
**C16:0**	65.2 ^b^	7.4	89.7^a^	9.8	69.6^b^	11.7	90.1^a^	10.8	75.8^b^	8.2	91.2^a^	9.4
**C18:0**	24.9^b^	3.4	40.7^a^	4.8	29.2^b^	3.6	41.6^a^	6.0	28.7^b^	3.8	42.2^a^	4.9
**C16:1 n-7**	48.9^a^	5.6	48.6^a^	5.6	46.8^a^	7.5	48.9^a^	5.9	54.7^a^	7.0	48.2^a^	5.8
**C18:1 n-9**	94.1^a^	11.2	97.4^a^	7.5	94.9^a^	7.6	99.1^a^	10.0	107.0^a^	8.8	99.0^a^	9.1
**C18:1 n-7**	44.9^a^	4.5	47.4^a^	4.2	49.9^a^	5.2	46.5^a^	4.1	50.0^a^	3.5	47.2^a^	3.3
**C18:2 n-6**	0.1^a^	0.1	0.2^a^	0.1	0.1^a^	0.0	0.1^a^	0.1	0.1^a^	0.2	0.2^a^	0.3
**C18:1 t11**	ND^5,c^		ND^c^		ND^c^		ND^c^		3.4^b^	0.7	5.5^a^	1.2
**C18:2 c9t11**	ND^d^		ND^d^		1.9^a^	0.4	1.4^b^	0.3	2.7^a^	0.6	0.9^c^	0.1
**C18:2 t11t13**	ND^c^		ND^c^		9.4^b^	1.6	15.5^a^	1.2	ND^c^		ND^c^	
**C18:3 c9t11t13**	ND^c^		ND^c^		1.3^a^	0.3	0.7^b^	0.2	ND^c^		ND^c^	

Caco-2 cells were cultivated in serum-free medium during 7 days post-confluence and then incubated for 24 h in the presence of TVA or t11t13 CLA at 20 µmol/l in absence or presence of sterculic oil (SO; 300 µmol/l of sterculic acid^1^).

1 Final concentration in culture medium.

2 t11t13 CLA: t11t13 conjugated linoleic acid (C18:2 t11t13).

3 TVA: *trans*-vaccenic acid (C18:1 t11).

4 ND: not detected.

Values are means ± SD from 3 independent experiments, each performed in triplicate (N = 3, n = 3) and are expressed in ng of each fatty acid per µg of cell protein. Means in the same row without a common superscript letter differ (P<0.05).

In order to modulate the bioconversion of t11t13 CLA, we also incubated the Caco-2 cells during 24 h, concurrently with t11t13 CLA and with SO, well known to contain inhibitors of the D9D. We first verified that SO had no cytotoxic effects on the cells at this concentration (results not shown). The inhibitory effect of SO in this cell culture system was first checked through a 24 h incubation of the Caco-2 cells with TVA, which can be efficiently converted into c9t11 CLA by the D9D (Table 1). The extent of D9D activity was evaluated from the ratio between the cellular concentration of c9t11 CLA and the sum of TVA and c9t11 CLA concentrations. In the absence of SO, this ratio approximated 0.44, whereas it was limited to about 0.14 upon cultivation with SO. This lower conversion rate of TVA in the presence of SO was associated with a significantly higher cellular level in TVA and a significantly lower level in c9t11 CLA. When the cells were incubated in the presence of t11t13 CLA and SO, the cellular concentration of t11t13 CLA was significantly increased while the abundance of c9t11 CLA and c9t11t13 CLnA was significantly decreased. These data implicate D9D in the first step of the conversion pathway between t11t13 CLA and c9t11 CLA. Accordingly, no TVA could be detected in the cells incubated with t11t13 CLA, in the absence or even in the presence of SO.

### Effect of sterculic oil on saturated and monounsaturated fatty acids

Table 1 shows an increase in the level of saturated fatty acids (SFA) (myristic acid, C14:0; palmitic acid, C16:0 and stearic acid, C18:0) in all the conditions with SO. The increase in palmitic acid may be explained, at least partially, by its relatively high abundance in SO. Accordingly, the increase in stearic acid may be due to an elongation of the excess of palmitic acid. By contrast, the increase in myristic acid, which appears more limited, is likely to be a consequence of D9D inhibition by SO. In contrast, the cellular levels of monounsaturated fatty acids (MUFA) (palmitoleic acid, C16:1 n-7; oleic acid, C18:1 n-9 and *cis*-vaccenic acid, C18:1 n-7) were not increased in the presence of SO, suggesting again an inhibitory effect of SO. Finally, the level of linoleic acid (C18:2 n-6) did not change when fatty acids or SO were added. In control cells, no other fatty acid was detected. Indeed, when cultivated in a serum-free medium, only the fatty acids that cells can synthesize *de novo* were accumulated except for linoleic acid that accumulated as a result of uptake from the nutritive medium.

### Time course of accumulation and conversion of t11t13 CLA

**Figure 3:**
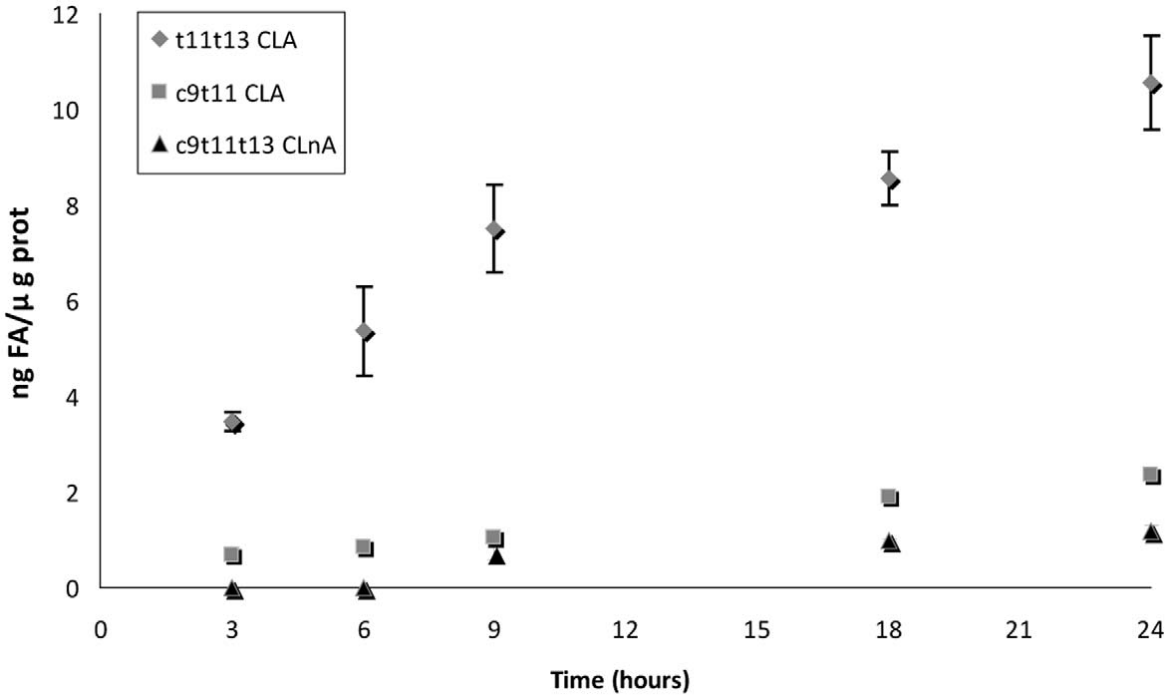
Time course of accumulation and processing of t11t13 CLA in Caco-2 cells. Caco-2 cells were cultivated in serum-free medium during 7 days post-confluence and then incubated with this fatty acid during 3 h, 6 h, 9 h, 18 h and 24 h at 20 µmol/l. Results are means ± S.D from 3 independent experiments, each performed in triplicates (N = 3, n = 3).

The time course of t11t13 CLA accumulation and metabolic processing into c9t11 CLA and c9t11t13 CLnA in Caco-2 cells was determined by addition of t11t13 CLA at 20 µmol/l for 3 h, 6 h, 9 h, 18 h or 24 h (Figure 3). The cellular level of t11t13 CLA increased rapidly up to 9 h (from 0 to about 7.5 ng/µg cell protein in 9 h) and then more slowly between 9 h and 24 h (to about 10.5 ng/µg cell protein in 15 h). The cellular level of the c9t11 CLA produced from t11t13 CLA increased with the incubation time up to 24 h. The accumulation kinetics of c9t11 CLA could be approximated by a linear regression (R^2^ of 0.995), which indicates a constant accumulation rate throughout the one-day incubation. Finally, the c9t11t13 CLnA intermediate also accumulated progressively but in lower proportions. It could be detected from 9 h on and its cellular level increased up to 24 h. According to the previous results, TVA could not be detected at any time in the cells incubated with t11t13 CLA during this one-day incubation, in the absence or even in the presence of SO.

### Distribution into lipid classes

**Figure 4:**
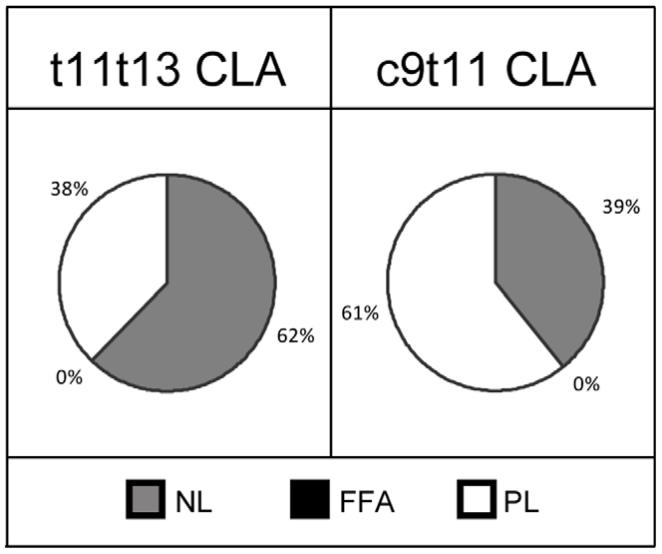
Incorporation of t11t13 CLA and its metabolite c9t11 CLA into different lipid fractions. Lipid fractions (neutral lipids (NL), free fatty acids (FFA) and phospholipids (PL)) were prepared from Caco-2 cells cultivated in serum-free medium during 7 days post-confluence and then incubated with t11t13 CLA (20 µmol/l) for 24 h. Results are means from 3 independent experiments (N = 3, n = 1).

The distribution of the t11t13 CLA accumulated by the Caco-2 cells, as well as that of its bioconversion product, c9t11 CLA, into the different lipid classes (NL, FFA and PL) was also determined (Figure 4). Unfortunately, the cellular level of c9t11t13 CLnA was too low to be detected when the cellular extract was divided in three fractions. It was first observed that t11t13 CLA as well as its metabolite, c9t11 CLA, were only distributed between the NL and PL fractions, since no fatty acid could be detected in the FFA fraction. Interestingly enough, the t11t13 CLA was found in a higher proportion in the NL fraction (62%), while the c9t11 CLA accumulated more readily in the PL fraction (61%).

## Discussion

Our results have shown that, upon incubation with t11t13 CLA, Caco-2 cells are able to absorb this conjugated fatty acid, but also to perform its metabolic processing into c9t11 CLA through the synthesis of c9t11t13 CLnA as intermediate. This latter fatty acid is probably produced from t11t13 CLA through the action of the D9D. The conversion of t11t13 CLA to c9t11 CLA has already been reported in a recent study using HT-29 cells [25]. To our knowledge, that study was the very first to show the conversion of one CLA isomer to another and our experiments in Caco-2 cells confirm these data. Furthermore, the data of the present study clarify the metabolic pathway involved in that conversion. Two metabolic routes presented in Figure 1 were plausible candidates, the first using TVA as intermediate and the other using c9t11t13 CLnA as intermediate. In their experiment using HT-29 cells incubated with t11t13 CLA, Degen *et al.* [25] observed only the presence of t11t13 CLA and of c9t11 CLA, and could unfortunately not detect the presence or accumulation of one of the two potential intermediate compounds. They were thus unable to draw conclusions about the chronology of action of the two enzymes required. The results of the present study definitively show that c9t11t13 CLnA is an intermediate involved in the conversion of t11t13 CLA to c9t11 CLA in Caco-2 cells. In addition, the inhibition of D9D by SO decreased the cellular level of c9t11t13 CLnA, confirming the desaturation of t11t13 CLA as being involved in the conversion pathway. By contrast, TVA (the other possible intermediate) was not detected, even in the presence of SO, which would have caused an increase in its concentration. This observation suggests the TVA route as being minor if not absent.

Both hypothetical metabolic pathways (Figure 1) involve the action of a D9D for the introduction of a double bond in the Δ9 position and of a reductase to saturate the fatty acid in the Δ13 position. D9D is a well-known enzyme with a broad substrate range [41] including TVA [16]–[21]. Among others, TVA was shown to be partly converted into c9t11 CLA in Caco-2 cells [42]. We confirmed these results in the present study. In addition, we showed a potent inhibitory effect of SO on that desaturation since a significantly higher cellular level of TVA and a significantly lower cellular level of c9t11 CLA were found in the presence of SO. The reductase activity targeting the Δ13 position is much less documented. It is know that c9t11t13 CLnA present in tung oil or in bitter gourd seed oil can be converted into c9t11 CLA in the liver, kidney and small intestine of rats and mice fed with these oils [28]–[31]. We have very recently shown that c9t11t13 CLnA can be reduced to c9t11 CLA in Caco-2 cells, in the same culture conditions as in this present study [33]. The enzyme involved in this conversion and the mechanism of action are however still not clearly identified. Tsuzuki *et al*. [28], [29] showed that c9t11t13 CLnA and c9t11c13 CLnA were saturated at the Δ13 position by a NADPH-dependent enzyme and converted into c9t11 CLA. The authors speculated that this Δ13 saturation was carried out either by an unknown enzyme recognizing conjugated trienoic acids or by the enzyme active in the leukotriene B4 reductive pathway, namely leukotriene B_4_ 12-hydroxydehydrogenase also named 15-ketoprostaglandin Δ13-reductase. Interestingly, Moise *et al*. [43], [44] recently described a retinol saturase, which carries out the saturation of the Δ13 double bond of the all-*trans*-retinol to produce all-*trans*-13,14-dihydroretinol. This enzyme may thus be another candidate for the Δ13 reduction of c9t11t13 CLnA. The fact that no TVA was detected in cells incubated with t11t13 CLA and SO suggests that the Δ13-reductase active on the fatty acids under investigations can not act on a fatty acid without a double bond in the Δ9 position.

A broader analysis of the cellular fatty acid profiles (Table 1) suggests that SO also influenced the synthesis of some fatty acids constantly produced *de novo* by the cells, even though its presence was limited to the last 24 h of incubation. This effect is visible for myristic acid, which is not present in SO. Its cellular accumulation is indeed significantly higher in the conditions with SO. For palmitic and stearic acids, the situation is less clear since palmitic acid may have been accumulated from SO added to the medium and then partly converted to stearic acid.

The t11t13 CLA was efficiently absorbed by the Caco-2 cells and this absorption was proportional to the duration of incubation for the first 9 h, and then slowed down. By contrast, accumulation of the conversion product c9t11 CLA, appeared to follow a linear kinetics during the whole experimental period of 24 h. As for the c9t11t13 CLnA intermediate, its accumulation pattern was again different. Indeed, no c9t11t13 CLnA was detected before 9 h of incubation and from this moment, its level increased slowly. Taken together, these observations suggest that the kinetics of the two enzymes involved are influenced differently in the range of concentrations encountered in the cells in the present experiment.

The distribution of t11t13 CLA and its bioconversion product, c9t11 CLA, into the different lipid classes (NL, FFA and PL) accumulated by the Caco-2 cells revealed intriguing differences. Unfortunately, the level of c9t11t13 CLnA was too low to be detected when the cellular extract was divided into the three fractions. The distribution of these CLA isomers was different but in both cases they were only distributed between the NL and PL fractions. We observed that the bulk of t11t13 CLA was found in the NL fraction while the isomer c9t11 CLA was principally found in the PL fraction. This difference in distribution could result from either the number of *trans* double bonds (one for c9t11 CLA and two for t11t13 CLA), the position of the double bonds (9,11 versus 11,13) or a combination of both. We have previously shown that the distribution between NL and PL fractions of four CLnA, differing only in terms of *cis* or *trans* configuration of their three double bonds, was correlated to the number of *trans* double bonds [33]. The same might be true here. Furthermore, we also showed that the relative position of the *trans* double bonds in the four CLnA bearing insaturations at the three same positions (9,11 and 13) seemed without influence on the repartition between the NL and PL fractions. Indeed, c9t11t13 CLnA and t9t11c13 CLnA were identically distributed between these two fractions. In that previous study, the distribution of c9t11 CLA between NL and PL was of 33–38% and 62–67%, respectively [33]. Similar results are obtained in the present study (61% in PL and 39% in NL), suggesting that the distribution of c9t11 CLA is independent from the substrate it originates: t11t13 CLA or CLnA. In contrast to the similarities in distribution for c9t11 CLA in the present study and in the previous one [33], differences appear if we compare the distribution of t11t13 CLA in the present study (38% in PL and 62% in NL) with that of t9t11 CLA in the previous study [33] (53–55% in PL and 45–47% in NL), suggesting that, in addition to the number of *trans* double bonds, their position does influence the cell fatty acid distribution pattern between PL and NL.

In conclusion, the present results tend to confirm that human enterocytes represented here by Caco-2 cells are able to convert one isomer of CLA into another one. In addition, they indicate that during the conversion of t11t13 CLA into c9t11 CLA, t11t13 CLA undergoes first a desaturation step catalysed by the D9D and then a reduction step targeting the double bond at the Δ13 position. We suggest here that this second enzyme is the same as the enzyme responsible of the conversion of CLnA into CLA.
